# Cellular Sources and Neuroprotective Roles of Interleukin-10 in the Facial Motor Nucleus after Axotomy

**DOI:** 10.3390/cells11193167

**Published:** 2022-10-09

**Authors:** Elizabeth M. Runge, Deborah O. Setter, Abhirami K. Iyer, Eric J. Regele, Felicia M. Kennedy, Virginia M. Sanders, Kathryn J. Jones

**Affiliations:** 1 Department of Anatomy, Cell Biology, and Physiology, Indiana University School of Medicine, Indianapolis, IN 46202, USA; 2 Research and Development, Richard L. Roudebush VA Medical Center, Indianapolis, IN 46202, USA; 3 Department of Physical Medicine and Rehabilitation, University of Pittsburgh School of Medicine, Pittsburgh, PA 15213, USA; 4 Department of Neurology, Mayo Clinic College of Medicine and Science, Rochester, MN 55905, USA; 5 Department of Psychiatry, Washington University School of Medicine, St. Louis, MO 63110, USA; 6 Department of Urology, Mayo Clinic College of Medicine and Science, Jacksonville, FL 32224, USA; 7 Department of Cancer Biology and Genetics, The Ohio State University, Columbus, OH 43210, USA

**Keywords:** astrocyte, axotomy, CD4+ T cell, facial nerve, IL-10, motoneuron, nerve injury, neuroprotection

## Abstract

Facial motoneuron (FMN) survival is mediated by CD4+ T cells in an interleukin-10 (IL-10)-dependent manner after facial nerve axotomy (FNA), but CD4+ T cells themselves are not the source of this neuroprotective IL-10. The aims of this study were to (1) identify the temporal and cell-specific induction of IL-10 expression in the facial motor nucleus and (2) elucidate the neuroprotective capacity of this expression after axotomy. Immunohistochemistry revealed that FMN constitutively produced IL-10, whereas astrocytes were induced to make IL-10 after FNA. *Il10* mRNA co-localized with microglia before and after axotomy, but microglial production of IL-10 protein was not detected. To determine whether any single source of IL-10 was critical for FMN survival, Cre/Lox mouse strains were utilized to selectively knock out IL-10 in neurons, astrocytes, and microglia. In agreement with the localization data reflecting concerted IL-10 production by multiple cell types, no single cellular source of IL-10 alone could provide neuroprotection after FNA. These findings suggest that coordinated neuronal and astrocytic IL-10 production is necessary for FMN survival and has roles in neuronal homeostasis, as well as neuroprotective trophism after axotomy.

## 1. Introduction

A robust peripheral immune response and the anti-inflammatory cytokine interleukin-10 (IL-10) are critical for maximal facial motoneuron (FMN) survival after peripheral nerve injury [1,2]. After facial nerve axotomy (FNA), immunodeficient mice lacking the adaptive arm of the immune system have decreased FMN survival in the facial motor nucleus (FMNuc), compared to wild-type (WT) mice [2,3]. The immune-mediated neuroprotective effects are specific to CD4+ T cells, which must encounter the antigen displayed on the major histocompatibility complex class II both peripherally and within the central nervous system (CNS) to confer neuroprotection [4,5]. The migration of CD4+ T cells into the CNS is mediated by centrally derived chemokines [6,7,8] and characterized by active extravasation of CD4+ T cells out of penetrating blood vessels and into the CNS parenchyma, where they may make direct contact with neurons and glial cells [9].

IL-10 is necessary for CD4+ T cells to mediate central FMN survival, but the precise role of IL-10 in the FMNuc has not been fully characterized. CD4+ T cells are not a requisite source of IL-10 in the injured FMNuc [1], but they lose their ability to confer neuroprotection when they lack a functioning IL-10 receptor (IL-10R). It is thought that a central cell type in the FMNuc produces IL-10, which promotes a tolerized CD4+ T cell response, discouraging a harmful form of microglial activation. While IL-10 is critical for FMN survival, previous data from our laboratory has shown that levels of IL-10 protein do not change significantly in response to axotomy [1]. The However, the presence of CD4+ T cells also promotes the upregulation of IL-10R on central cells in the FMNuc, which may have further trophic and/or auto-inflammatory roles that promote neuronal survival [9]. This supports the hypothesis that there is an increase in neuroprotective IL-10 signal-ing events regardless of the cytokine’s relatively static quantity after axotomy. The cellular source of IL-10 nonetheless remains a critical component of this cascade. Yet despite the evidenced importance of centrally derived IL-10 in regulating the CD4+ T cell and glial responses to axotomy, the precise cellular source of neuroprotective IL-10 in this system has not been previously identified.

The major aim of this study was to determine the central cellular sources of IL-10 in the facial motor nucleus and the respective contributions of these sources to FMN survival after axotomy. After ruling out a peripheral immune contribution of neuroprotective IL-10, the first part of this aim was tested using two methods of immunohistochemical (IHC) identification of IL-10-producing cells, as well as fluorescent in situ hybridization (FISH) for *Il10* mRNA in the FMNuc. The results revealed that FMN produce IL-10 constitutively, whereas astrocytic IL-10 expression is axotomy-induced. *Il10* mRNA was detected within microglia, but protein expression was not evident using these experimental methods. For the second part of this aim, IL-10 was selectively knocked out in neurons, astrocytes, and microglia using Cre/Lox technology. In agreement with the localization data, no single cellular source of IL-10 alone conferred neuroprotection. These data lead to the conclusion that multiple central sources of IL-10, perhaps with redundant roles, coordinate to promote FMN survival after axotomy.

## 2. Materials and Methods

### 2.1. Animals

One can refer to Table 1 for a list of the animal strains used in this study. For the animals obtained from The Jackson Laboratory, only female mice were used due to the tendency of male non-littermates to fight after axotomy, leading to infections at the surgical site. Animals were obtained at six or seven weeks of age and acclimated undisturbed for at least one week. For animals bred in house (IL10^flox/flox^ strain crossed with all Cre-expressing strains), equal numbers of male and female mice were used in the experiments. All animal procedures complied with the National Institutes of Health guidelines on the care and use of laboratory animals and were approved by the Institutional Animal Care and Use Committee at the Indiana University School of Medicine (protocol #11227).

### 2.2. Preparation of Genomic DNA and Polymerase Chain Reaction (PCR)

Genomic DNA was extracted from the tail snips (for genotyping) and spleen (to confirm adoptive transfer engraftment) using the Gentra^®^ PureGene^®^ Core Kit A (QIAGEN Sciences, Germantown, MD, USA, cat. No. 1042601) following the manufacturer’s instructions. PCR was performed on genomic DNA using the primers listed in Table 2 and illustra™ PuReTaq Ready-To-Go ™ PCR Beads (GE Life Sciences, cat. No. 27955701). All IL10 PCR was performed using the following program: 95 °C for 2 min; 30 cycles of 95 °C for 30 s, 60 °C for 45 s, 72 °C for 45 s; and 72 °C for 5 min. All Cre PCR was performed using the following program: 94 °C for 2 min; 10 cycles of 94 °C for 20 s, 65 °C for 15 s with −0.5 °C per cycle decrease, 68 °C for 10 s; 28 cycles of 94 °C for 15 s, 60 °C for 15 s, 72 °C for 10 s; and 72 °C for 2 min. PCR amplicons were run on a 1.5–2% agarose gel at 120–150 V for 20–40 min and were subsequently imaged using a Cell Biosciences FluorChem E system. 

### 2.3. Adoptive Transfer

Donor animals were utilized at a 1:1 ratio to recipients. Following CO_2_ euthanasia and cervical dislocation, the spleens were removed and mechanically dissociated with the back of a sterile syringe in 1–2 mL of cold working buffer (0.5% BSA + 2 mM EDTA in 1X PBS). Cells were filtered, pelleted, and resuspended in 1 mL of red blood cell lysis buffer (150 mM NH_4_Cl + 10 mM KHCO_3_ + 0.1 mM EDTA in ddH_2_O) and left to rest for 5 min at room temperature prior to washing and counting in the working buffer. Cells were resuspended in a suitable volume of 1X PBS to yield 50 × 10^6^ whole splenocytes per 100 μL injection, which was performed via the tail vein one week prior to FNA.

### 2.4. Induction of Cre Recombinase

Animals expressing at least one copy of Cre recombinase and homozygous for floxed IL-10 sequences (Cre^+/+^IL10^fl/fl^ or Cre^+/−^IL10^fl/fl^) were used in the experimental group. Animals negative for either Cre or floxed IL-10 sequences (Cre^−/−^IL10^any^ or Cre^any^IL10^wt/wt^) were used as littermate controls. One week prior to axotomy (seven weeks of age), animals in both groups were given once daily intraperitoneal injections of 75 mg/kg tamoxifen (Sigma-Aldrich, St. Louis, MO, USA, cat. No. T5648-5G) suspended in corn oil (Sigma-Aldrich, St. Louis, MO, USA, cat. No. C8267), at a concentration of 20 mg/mL for five consecutive days. To maintain Cre expression, injections were administered on a biweekly basis until euthanasia. 

To evaluate the extent of IL-10 knockdown, the mice were given once daily intraperitoneal injections of tamoxifen for five consecutive days, followed by one week of biweekly injections. Two weeks after the tamoxifen induction of Cre was started, LPS was injected at 5 mg/kg to stimulate IL-10 production in glia. LPS was not administered to the animals used for the evaluation of neuronal IL-10 production. Animals were euthanized at either 3 h (for evaluation of microglia) or 12 h (for astrocytes) post LPS injection.

Following euthanasia and brain removal, brains were dissociated using the MACS Neural Tissue Dissociation Kit (P) (Miltenyi Biotec, cat. No. 130-092-628), following the manufacturer’s protocol for manual dissociation, and cells were sorted using anti-CD11b (Miltenyi Biotec, cat. No. 130-093-634), anti-GLAST (130-098-803), or neuron isolation kit (130-115-390) microbeads, per the manufacturer’s instructions. Sorted cells were pelleted, washed with PBS, and lysed with Pierce ™ RIPA Lysis and Extraction Buffer (Thermo Scientific™, Waltham, MA, USA, cat. No. 89900). Protein concentration was determined using a Pierce™ BCA Protein Assay Kit (Thermo Scientific™, Waltham, MA, USA,). For evaluation of IL-10 in glia, 25–100 ng protein was blotted onto a nitrocellulose membrane and probed with a rat anti-mouse IL-10 antibody (Invitrogen, Waltham, MA, USA, cat. No. 12-7101-81) at 1:1000 dilution, followed by goat anti-rat HRP (Abcam, Cambridge, UK, cat. No. ab97057) at 1:25,000 dilution. HRP signal was detected using GE Healthcare Amersham™ ECL™ Prime Western Blotting Detection Reagent (Fisher Scientific, Rockingham County, NH, USA, cat. No. 45-002-401), following the manufacturer’s instructions. Integrated density measurements were performed on dot blot images in ImageJ. IL-10 in microglia was determined to be 14–34% reduced in cKO compared to the littermate controls, and IL-10 in astrocytes was determined to be 51–83% reduced (data available upon request). For evaluation of IL-10 in neurons, protein lysates were analyzed using a Mouse IL-10 ELISA MAX™ Deluxe kit (BioLegend, San Diego, CA, USA, cat. No. 431414). IL-10 was reduced by approximately 27% in the neurons isolated from cKO animals compared to the littermate controls (data available upon request).

### 2.5. Facial Nerve Axotomy

For ease of visualization, the timelines for each experiment in relation to the day of FNA is summarized in Supplementary Figure S1. FNA was performed when the mice were eight weeks old. The detailed surgical procedure has been published previously [11]. Briefly, the animals were anesthetized with 2.5% isoflurane prior to surgical site preparation using aseptic techniques. A small incision was made posterior to the ear protuberance, and the facial nerve was exposed by blunt dissection through the underlying fascia and severed just distal to its emergence from the stylomastoid foramen. Transection of the facial nerve was confirmed post-surgically by absence of the eye blink and whisking reflexes.

### 2.6. Motoneuron Counting

The protocol for motoneuron counting has been described previously [9]. Briefly, animals (n = 3–8) were euthanized at 28 dpo (days post-operation) using CO_2_ inhalation and cervical dislocation. Brains were removed, flash frozen in a biphasic 2-methylbutane/1-bromobutane solution, and cryosectioned at 25 μm prior to fixation in paraformaldehyde (PFA) and staining with thionin acetate. The entire FMNuc was collected, sectioned, counted, and included in the analysis for each animal.

An impartial investigator coded all the slides prior to counting by a separate investigator, which was performed manually for all FMN profiles, with a nucleus and nucleolus spanning the entire rostro-causal extent of the FMNuc using a Leica DMRB light microscope and Neurolucida software (version 10.31). Mean percentage FMN survival was quantified by comparing the axotomized side to the intact control side. A Student’s *t*-test was performed for experiments containing ≤2 groups, and one-way analysis of variance (ANOVA) followed by a Student–Newman–Keuls post-hoc test was performed for experiments containing ≥2 groups with an alpha of 0.05.

### 2.7. Immunohistochemistry

#### 2.7.1. IL-10/GFP Reporter

Determination of the optimum conditions for the detection of GFP in the IL-10/GFP reporter mouse revealed a detrimental effect of extensive PFA fixation on the ability to detect GFP signal (data not shown). It is possible that perfusion fixation denatured the GFP structure, which is a known issue when working with PFA [12]. As both IL-10 and GFP expression in the reporter mouse are controlled by the endogenous IL-10 promoter, and thus are likely to be present at relatively low levels, the presence of aldehyde residue induced-autofluorescence may also obscure the GFP signal [13,14]. Therefore, to preserve GFP labeling, the brains (n = 3/time point) were flash-frozen immediately after removal (see Section 2.6) and lightly post-fixed on the slide as described below, thereby avoiding potential over-fixation due to perfusion-based methods. Various time points (3, 7, 10, 14, and 28 dpo) were studied. Brainstem sections spanning the caudal-rostral extent of the FMNuc were cryosectioned at 8 μm, collected onto Superfrost Plus slides (Thermo Scientific, Waltham, MA, USA), and stored at −20 °C. Sections were post-fixed in 4% PFA for 5 min and blocked in 10% donkey serum, 1% BSA, and 0.01% Triton X-100 in PBS for one hour prior to incubation, with primary antibodies (Table 3) diluted in the same blocking medium overnight at 4 °C, followed by washing and incubation with the secondary antibody for 1 h at room temperature. Slides were washed and mounted with DAPI Fluoromount-G^®^ (Southern Biotech, Birmingham, AL, USA, cat. No. 0100-20). Images were captured with an Olympus BX50 inverted fluorescent microscope using Olympus cellSens Entry 1.9 software, and brightness/contrast adjustments to reduce background were performed in Adobe Photoshop.

#### 2.7.2. Immunohistochemistry Performed on Wild-Type Tissue

The animals (n = 3/time point) were euthanized at 3-, 7-, 10-, 14-, and 28-day time points via ketamine-xylazine overdose and exsanguination and perfused with 2% buffered PFA. Brains were removed, post-fixed in 2% PFA for 1 h, equilibrated in 30% sucrose, and embedded in OCT medium. The 8 μm brainstem sections containing the FMNuc were blocked as described in Section 2.7.1, followed by incubation in the primary antibody (Table 3) for 2 h at room temperature or 16 h at 4 °C. NeuroTrace™ 435/455 Blue Fluorescent Nissl Stain (Thermo Fisher, Waltham, MA, USA, cat. No. N21479) was diluted 1:100 in PBS, applied to sections for 20 min after removing the secondary antibody, and washed with PBS prior to mounting in ProLong™ Gold Antifade medium (Invitrogen, Waltham, MA, USA, cat. No. P36930). Imaging proceeded as described in Section 2.7.1.

### 2.8. Fluorescent In Situ Hybridization

The animals (n = 3/time point) were euthanized, and brains were removed, flash-frozen, and sectioned at 8 μm, as described in Section 2.6. FISH was performed using Stellaris^®^ RNA FISH probes (LGC Biosearch Technologies, Hoddesdon, UK), following the manufacturer’s instructions that are available online at www.biosearchtech.com/stellarisprotocols with the following changes. Sections were fixed for 4 h in 4% PFA, washed 2 × 10 min in PBS, dipped in H_2_O, and equilibrated in 1X triethanolamine (TEA) buffer for 10 min. Next, slides were immersed in 1X TEA + 0.25% acetic anhydride for 10 min (stirring) and washed in 2X SSC for 10 min. Slides were then dehydrated following the Stellaris^®^ protocol. After air drying, slides were hybridized with 1000 nM Stellaris^®^ RNA FISH probes diluted in Stellaris^®^ hybridization buffer in a humidified chamber at 55 °C overnight. Ready-made probes were used for *Cx3cr1* Quasar^®^ 570 (cat. No. VSMF-3102-5), and custom probes for *Il10* Quasar ® 670 were designed using the Stellaris ® FISH Probe Designer for sequence NM_010548.2. Day 2 of the protocol followed the manufacturer’s instructions, with the exception of using DAPI Fluoromount-G^®^ (Southern Biotech, Birmingham, AL, USA) mounting medium, instead of separate DAPI labeling and slide mounting. Imaging was performed using a Nikon A1 confocal microscope, and post-processing to remove the background was performed in ImageJ.

## 3. Results

### 3.1. Contribution of IL-10 from Peripheral Immune Cells to Central Facial Motoneuron Survival

**Figure 1:**
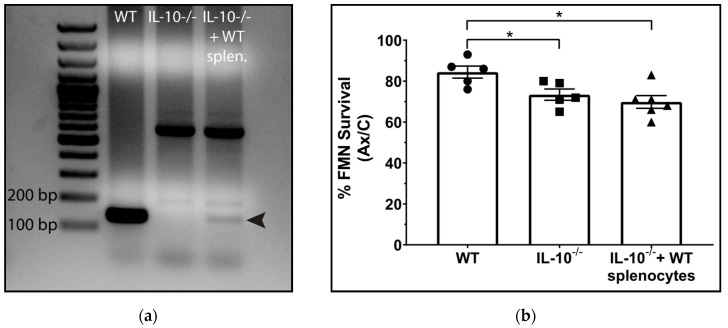
(**a**) PCR amplicon gel showing a 133 bp fragment (arrow) of exon 1 of the IL10 coding sequence, which is present in WT, absent in IL-10^−/−^, and restored in IL-10^−/−^ reconstituted with WT whole splenocytes. (**b**) Average percent survival of axotomized FMN relative to the control ± SEM at 28 dpo. No significant difference was detected between IL-10^−/−^ and IL-10^−/−^ receiving splenocytes; survival in both groups was significantly decreased relative to WT (* *p* < 0.05). WT *n* = 5, IL-10^−/−^ *n* = 5, IL-10^−/−^ + WT splenocytes *n* = 6.

Previous studies have shown that although IL-10 is necessary for CD4+ T cells to mediate neuroprotection, CD4+ T cells themselves are not the required IL-10 producers in this injury model [1]. Prior to operating under the assumption that the source of neuroprotective IL-10 must be centrally located, it was first essential to eliminate other peripheral sources of IL-10, such as antigen presenting cells (APCs), as potential candidates. This is due to the possibility that IL-10 produced by peripheral APCs in the lymph nodes may be necessary for CD4+ T cells to differentiate to a neuroprotective subtype after axotomy. FNA was performed on WT, IL-10^−/−^, and IL-10^−/−^ mice reconstituted with WT whole splenocytes (a heterogeneous population of T cells, B cells, and APCs). Successful engraftment of the WT splenocytes was confirmed by performing PCR on genomic DNA extracted from WT, IL-10^−/−^, and reconstituted IL-10^−/−^ spleens, using primers flanking a 133 bp sequence within exon 1 of the IL10 gene. In the IL-10^−/−^ animal, codons 5-55 of exon 1 are replaced with a termination codon, followed by a neomycin cassette [15]. Gel electrophoresis of the resulting PCR amplicons revealed that the 133 bp fragment was present in WT, absent in IL-10^−/−^ mice, and restored in the IL-10^−/−^ mice reconstituted with WT whole splenocytes, indicating that transplanted WT splenocytes were capable of engrafting into host lymphatic tissue, and furthermore survived within the host until at least 28 dpo, when FMN survival was quantified (Figure 1a). FMN survival in IL-10^−/−^ mice (Figure 1b, 73.4 ± 2.77%) as well as in WT whole splenocyte-reconstituted IL-10^−/−^ mice (69.8 ± 3.11%) was significantly reduced compared to WT (84.4 ± 2.94%, *p* = 0.01), indicating that IL-10 produced by any peripheral immune source is not sufficient for FMN survival after axotomy. 

### 3.2. IL-10/GFP Reporter Characterization

With a peripheral immune contribution of neuroprotective IL-10 effectively ruled out, the detection of the central source of IL-10 was pursued. A transgenic IL-10/GFP reporter mouse line was utilized to detect the central sources of IL-10. This line was created by Dr. Christopher Karp’s laboratory via the insertion of an internal ribosome entry site (IRES) downstream of exon 5 of the *Il10* locus [14]. Transcription of the resultant IL-10/GFP fusion mRNA occurs under the control of the endogenous IL-10 promoter, but separate IL-10 and GFP proteins are translated. The result is independent, yet spatiotemporally coupled translation of IL-10 and GFP proteins, the former capable of being secreted from the producing cell, and the latter remaining behind to label the cell body.

**Figure 2:**
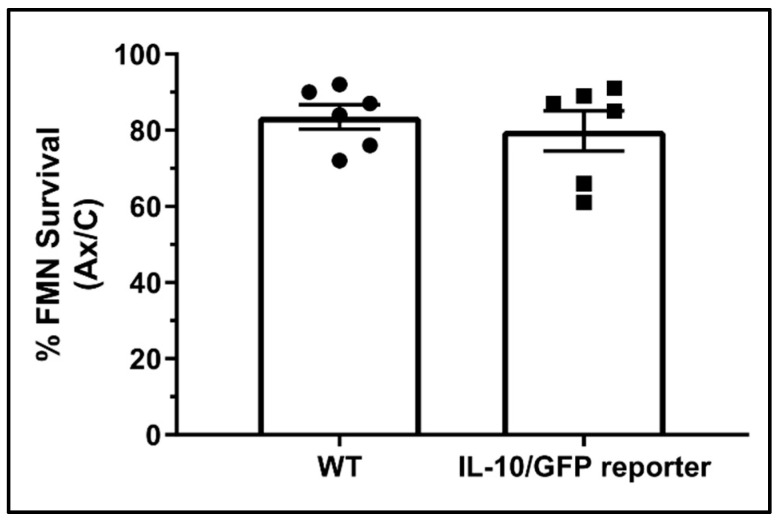
FMN survival in WT and IL-10/GFP reporter mice. Average percent survival of axotomized FMN relative to the control ± SEM at 28 dpo. No significant difference was detected between WT and reporter (*n* = 6 for both groups).

To confirm that the IL-10/GFP reporter represents a biological system capable of synthesizing and secreting functional IL-10 in a manner similar the WT animal, FNA was performed on WT and IL-10/GFP mice. Quantification of FMN survival at 28 dpo revealed no significant difference between the WT and transgenic reporter (Figure 2, 83.5 ± 3.24% and 79.83 ± 5.27%, respectively, *p* = 0.57), indicating that IL-10 was functioning normally in the IL-10/GFP mice.

### 3.3. Central Cellular Localization of IL-10

Neurons, microglia, and astrocytes were investigated as potential sources of IL-10 in the axotomized FMNuc. It was hypothesized that microglia are the major central cellular source of IL-10, as microglia are capable of producing IL-10 in vitro [16,17], particularly upon interacting with T cells [18], as well in vivo in response to CNS injury [19]. The cellular source of IL-10 was investigated in the FMNuc at 3, 7, 10, 14, and 28 dpo.

#### 3.3.1. Neurons

**Figure 3:**
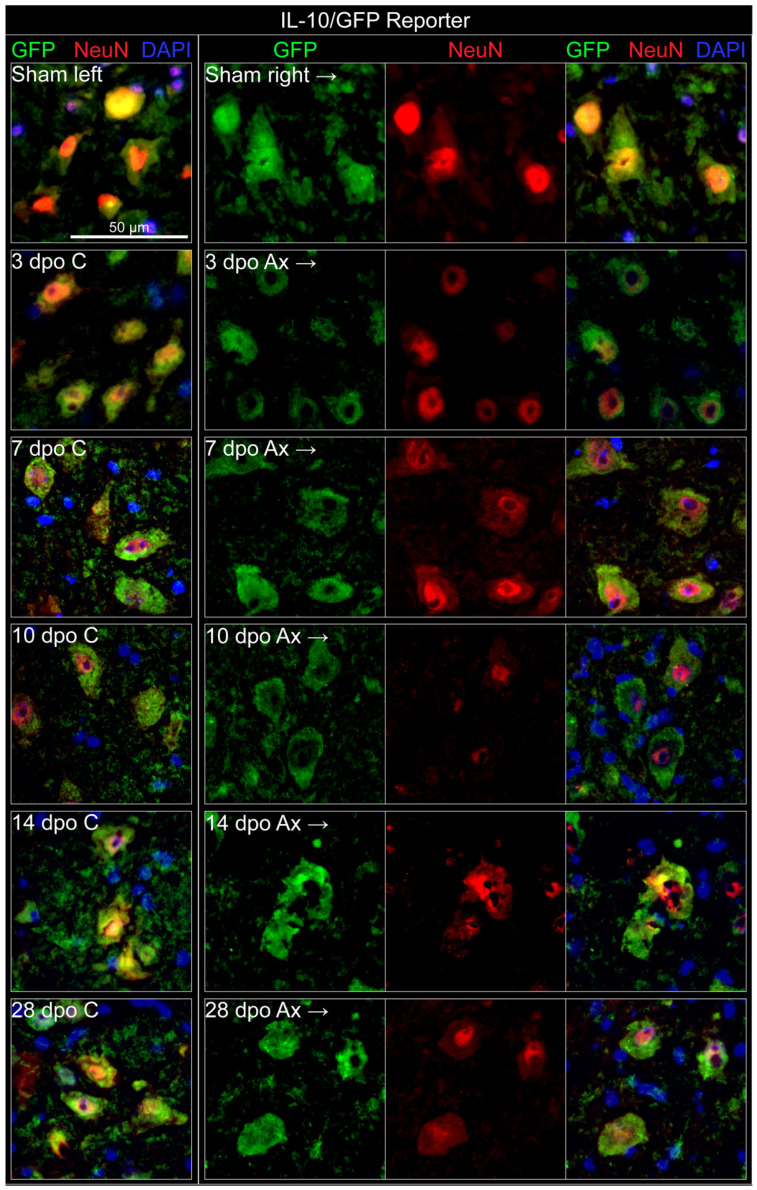
IHC co-localization of neurons (NeuN) with GFP in IL-10/GFP reporter mouse. Dpo = days post operation, C = control (**left**) FMNuc, Ax = axotomized (**right**) FMNuc.

Neuronal production of IL-10 was investigated utilizing IHC co-labeling of GFP (as a proxy for IL-10 production), with NeuN (neuronal nuclei marker) in the IL-10/GFP reporter mouse. NeuN is associated with a mature neuronal phenotype and becomes less immunoreactive after axotomy [20,21]. This effect can be appreciated in Figure 3, particularly at 10 dpo. GFP labeling was detected in both the left and right FMNuc of the unoperated (sham) animal, as well as in the control and axotomized FMNuc of operated animals at all time points investigated (Figure 3). Across all conditions, GFP localized strongly to the cytoplasm of cells with NeuN-labeled nuclei. This GFP-neuronal co-localization did not change appreciably, regardless of axotomized condition or time point, indicating that FMN may be a constitutive source of IL-10.

#### 3.3.2. Microglia

A microglial source of IL-10 was next investigated utilizing the marker IBA1, which is specific to microglia in the CNS [22,23]. Although IBA1 reliably labels microglia in perfusion-fixed tissue, attempts to utilize the IBA1 marker in the flash-frozen IL-10/GFP reporter tissue failed to label microglia (data not shown). Antibody labeling for other microglia-associated markers, such as CD68 and F4/80, also failed to yield sufficient labeling of microglia, suggesting that microglial structures may be exquisitely sensitive to fixation methods.

**Figure 4:**
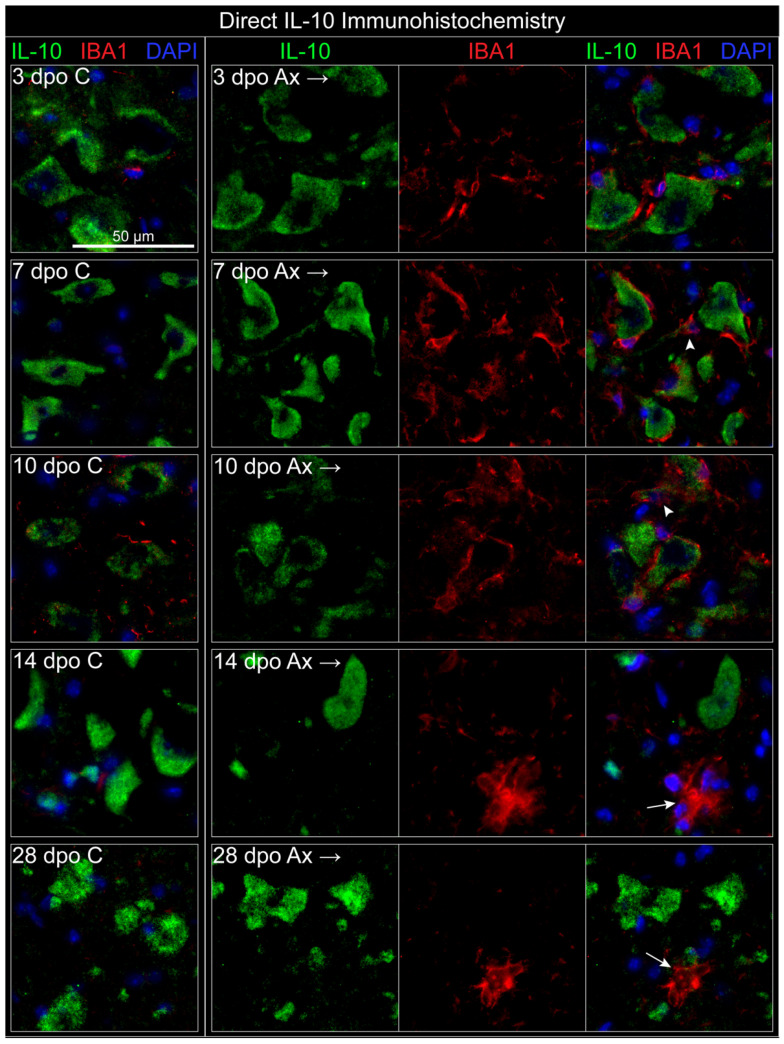
Direct IHC localization of microglia (IBA1) and IL-10 in WT mice. Arrowheads at 7 and 10 dpo demonstrate microglia lying in close proximity to IL-10-positive neuronal processes. Arrows at 14 and 28 dpo indicate microglial nodules. Dpo = days post operation, C = control (**left**) FMNuc, Ax = axotomized (**right**) FMNuc.

As an alternate method of identifying IL-10 production by microglia, IBA1 labeling was utilized alongside a direct antibody against IL-10 protein in WT tissue. This avoidance of the IL-10/GFP reporter permitted the use of perfusion fixation, which restored IBA1 labeling of microglia in the FMNuc (Figure 4). IL-10 antibody robustly labeled cells with a classic motoneuron morphology in both the control and axotomized FMNuc at all time points. This IL-10 labeling appeared to be restricted to the cytoplasm and left a central clearing occupied by DAPI, which intercalates with DNA and labels cell nuclei. These data support the previous conclusion utilizing the IL-10/GFP reporter mouse that neurons are constitutive producers of IL-10 in the FMNuc.

IBA1 labeling in the control FMNuc revealed small numbers of microglia processes and the occasional microglia cell body (see Figure 4, 3 dpo C). After axotomy, increased IBA1 immunoreactivity at 3, 7, and 10 dpo demonstrated microglia surrounding injured motoneuron cell bodies, which have been characterized as a sequela of synaptic stripping in prior studies [24,25]. Microglia appeared in close proximity to the FMN, and therefore also to FMN-associated IL-10 immunoreactivity, making discernment of neuronal- and microglial-associated expression of IL-10 difficult to distinguish. In some instances, microglia appeared to co-localize with the IL-10 signal (Figure 4, arrowheads at 7 and 10 dpo), but upon close observation, this apparent “co-localization” was likely to be due to the intimate association of microglia with neuronal processes. Most observed microglia appear not to co-localize with IL-10 labeling. At later time points (14 and 28 dpo), IBA1 labeled nodular structures, which are likely to be clusters of microglia phagocytizing debris from dead neurons (Figure 4, arrows) [25,26]. These clusters did not co-localize with IL-10 labeling.

**Figure 5:**
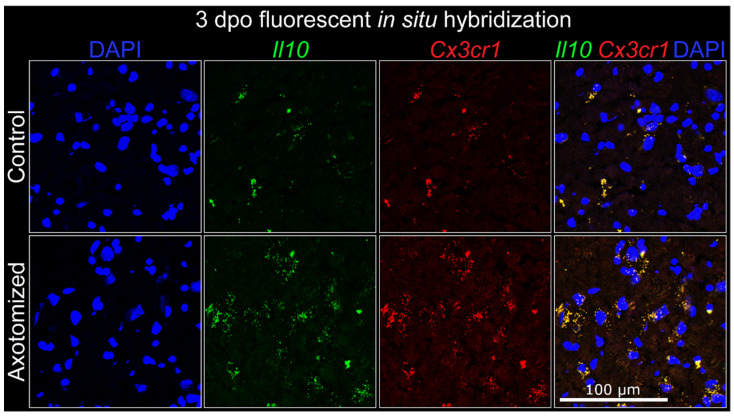
Fluorescent in situ hybridization for *Il10* and *Cx3cr1* mRNA transcripts.

Although IL-10 labeling of neurons appeared to be consistent between the IL-10/GFP reporter and IHC on WT tissue, antibody labeling of a secreted ligand, such as IL-10, presents several uncertainties. The particular IL-10 antibody utilized in this study binds at the IL-10R binding site, and therefore labels only free or intracellular IL-10 [27]. However, if a ligand is secreted rapidly, the antibody may not label the cellular source. Therefore, a second method of localization was desired to further elucidate whether microglia are a potential source of IL-10 in the FMNuc after axotomy. FISH was utilized to co-localize *Il10* and *Cx3cr1* transcripts in the facial motor nucleus at 3 days post injury. *Cx3cr1* encodes the fractalkine receptor, which is primarily expressed on microglia in the CNS and has important roles for intracellular calcium mobility and chemotaxis, particularly in response to neuronal damage [28]. FISH revealed the presence of scattered punctate areas of co-localized *Il10* and *Cx3cr1* probes in the control FMNuc, which appeared to increase in number at 3 dpo (Figure 5). This raises the possibility that microglia make *Il10* mRNA in the FMNuc, but whether this is translated into functional protein warrants further investigation.

#### 3.3.3. Astrocytes

**Figure 6:**
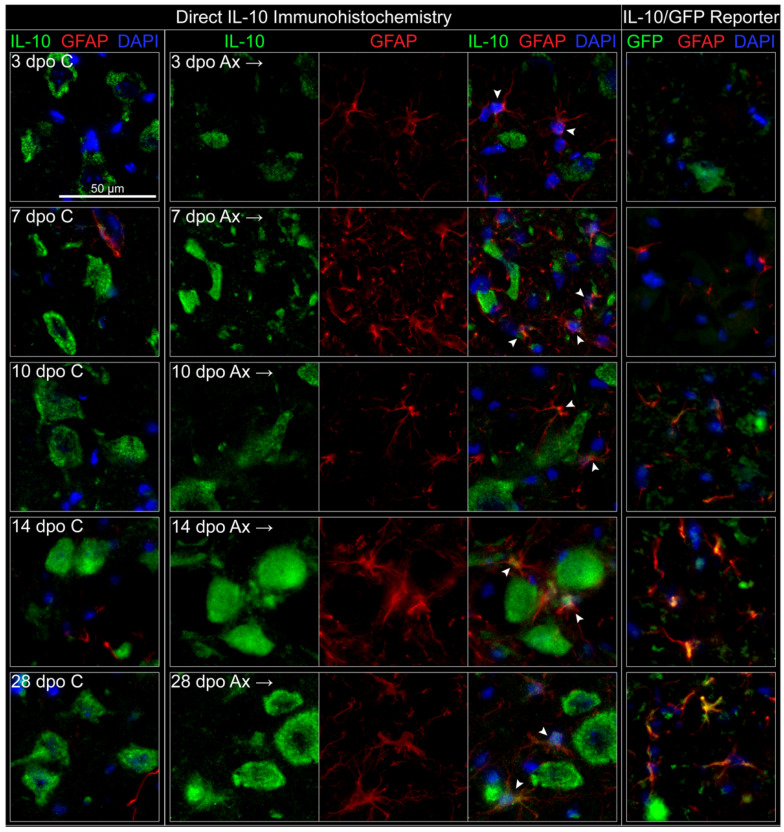
IHC co-localization of astrocytes (GFAP) with IL-10 using both direct IL-10 antibody and IL-10/GFP reporter. Left panels depict direct IL-10 antibody, right panels depict reporter. Arrowheads indicate areas of co-localization. Dpo = days post operation, C = control (**left**) FMNuc, Ax = axotomized (**right**) FMNuc.

To determine whether astrocytes are a source of IL-10 in the facial motor nucleus, IHC labeling of the astrocyte-associated intermediate filament protein GFAP was utilized. Because it was found that reliable GFAP labeling can be achieved using both perfusion-fixed and post-fixed flash frozen tissue, both the direct IHC method using IL-10 antibody on WT tissue, as well as GFP labeling of IL-10/GFP reporter, were utilized. Using direct IL-10 IHC, IL-10 again robustly labeled cells on both the control and axotomized FMNuc in a characteristic motoneuron-like pattern (Figure 6). GFAP labeling in the control FMNuc was mainly restricted to astrocytes lining capillaries and along the *glia limitans* on the ventral edge of the brainstem. GFP co-localized occasionally with GFAP-labeled astrocytes adjacent to blood vessels (Figure 6, 7 dpo C). GFAP-labeled astrocytes were detected within the FMNuc parenchyma in response to axotomy, beginning at 3 dpo and persisting through 28 dpo. Weak IL-10 co-localization with GFAP was detectable at early time points after axotomy (Figure 6, arrowheads at 3, 7, and 10 dpo) and increased in strength at later time points, as GFAP-labeled astrocytes became increasingly hypertrophied (arrowheads at 14 and 28 dpo). IL-10 labeling appeared mainly in astrocyte cell bodies, but occasionally also labeled processes.

The IL-10/GFP reporter also displayed a progressive increase in GFAP immunoreactivity in the time points following axotomy, but unlike in the perfusion-fixed WT tissue, GFAP-labeled astrocytes were scarce at 3 dpo (Figure 6, right pane). This delay in the onset of GFAP immunolabeling is likely attributable to the fixation method used to preserve the GFP signal in the reporter mouse. GFP labeling representing IL-10 production began to co-localize with GFAP by 10 dpo. By 14 and 28 dpo, nearly all GFAP-labeled astrocytes observed in the FMNuc were also positive for GFP. GFP labeling other structures in the FMNuc, likely motoneurons, could be observed for all the time points.

In summary, axotomy induced the expression of IL-10 by astrocytes. This expression was detectable beginning at early (3 dpo) time points when utilizing direct IL-10 antibody in perfused tissue, and at later (10 dpo) time points using the IL-10/GFP reporter. This expression appeared to persist through 28 dpo using both labeling methods. Neuronal expression of IL-10 appeared to be constitutive and did not noticeably change after axotomy. Microglial production of IL-10 was not detected by immunohistochemical methods, although there is evidence that microglia transcribe *Il10* mRNA.

### 3.4. Cell-Specific Conditional IL-10 Knockout

With at least two potential sources of IL-10 in the FMNuc identified, it was next important to determine whether any single source was necessary for FMN survival after axotomy. Three mouse strains were created to selectively knock out IL-10 expression in single central cell populations using tamoxifen-inducible Cre recombination of floxed IL-10 sequences. Cre recombinase expression was driven by the promoters for *Thy1*, *Cx3cr1*, and *Gfap* for targeted deletion of IL-10 in neurons, microglia, and astrocytes, respectively. The *Thy1*^Cre^ mouse contains a modified *Thy1* promoter region that restricts its expression to neuronal cells [29]. Tamoxifen induction of Cre began one week prior to axotomy and continued until 28 dpo, at which time FMN survival was assessed. The littermates lacking either Cre or floxed IL-10 sequences were used as controls and received identical treatments to experimental mice.

**Figure 7:**
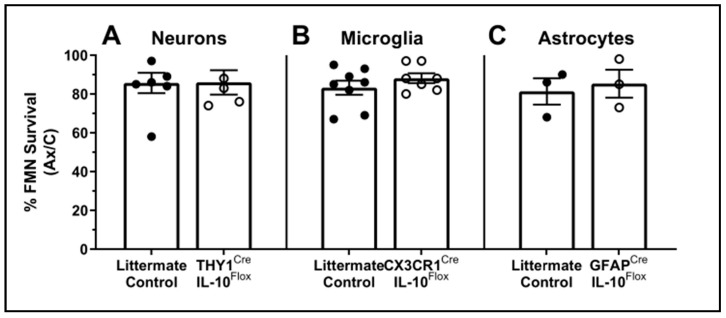
FMN survival in IL-10 cKO mice compared to littermate controls (Cre^−/−^ or IL-10^wt/wt^). Average percent survival of axotomized FMN relative to control ± SEM at 28 dpo in mice lacking IL-10 in (**A**) neurons (control *n* = 7, cKO *n =* 5), (**B**) microglia (control *n* = 8, cKO *n =* 7), and (**C**) astrocytes (*n* = 3 for both groups). No significant differences were observed between littermates and cKO.

Conditional knockout (cKO) of IL-10 did not significantly reduce FMN survival in any of the Cre-expressing mouse lines compared to the littermate controls, indicating that IL-10 production by neurons, microglia, or astrocytes alone was not critical for neuroprotection after axotomy (Figure 7, neurons: 85.7 ± 5.22% in control vs. 86 ± 6.27% in cKO, *p* = 0.97; microglia: 83.3 ± 3.65% vs. 88.1 ± 2.54%, *p* = 0.30; astrocytes: 81.3 ± 6.77% vs 85.3 ± 7.22%, *p =* 0.71, respectively). This is in agreement with the localization data showing IL-10 production by both neurons and astrocytes at minimum. It is likely that the conditional knockdown of IL-10 from one of these sources induces compensatory production by another.

## 4. Discussion

It is highly possible that infiltrating CD4+ T cells produce IL-10 in the FMNuc, but they are not a requisite source of IL-10 for neuroprotection after axotomy [1]; the neuroprotective capacity of CD4+ T cells relies on functions other than their endogenous IL-10 production, which is further discussed below. However, IL-10 production by other peripheral immune cells remained a potential alternative, given the ability of peripheral IL-10 to modulate T cell subset activation. This study found that WT splenocytes are incapable of supporting FMN survival in the IL-10^−/−^ animal after axotomy. Although this study did not quantify the exact proportion of surviving splenocytes, or their IL-10 production capability after adoptive transfer, the quantity and method selected for transfer of splenocytes in this study have been shown previously to be sufficient for establishing normal splenic follicular architecture [2] and rescuing FMN survival [3] in immunodeficient mouse models. These results support the hypothesis that a central source of IL-10 is critical for neuroprotection. IL-10 is produced constitutively by motoneurons in the FMNuc and is induced in astrocytes after axotomy. Microglia may constitutively transcribe *Il10* mRNA, but there is currently no evidence for translation of the IL-10 protein in microglia after FNA. Neuroprotective IL-10 in the FMNuc does not derive exclusively from any single cellular source investigated in this study (that is, neurons, astrocytes, or microglia), and it is likely that compensation can occur when production by one of these sources is lost.

The demonstration of constitutive IL-10 production by FMN was an unexpected discovery, given the relative dearth of studies in the literature showing IL-10 production by neurons compared to glia. An autocrine role for neuronal IL-10 is supported by the reports in vivo that FMN and healthy cortical, striatal, and hippocampal neurons express IL-10 and its receptor constitutively and/or under conditions of ischemic stress [1,30,31]. In vitro studies of primary neuron cultures have shown constitutive basal IL-10 production, which increases after prolonged toll-like receptor (TLR) stimulation [32], and application of an IL-10 neutralizing antibody in the absence of exogenous IL-10 promotes neuron apoptosis and suppresses the expression of synaptic components under stressful conditions [33]. These data suggest that endogenous IL-10 production by neurons may have an autocrine role in conferring resistance to ischemia and metabolic stress, as well as in homeostatic maintenance of synapses.

Astrocyte production of IL-10 has been characterized both in vitro and in vivo. Primary astrocytes constitutively produce low levels of IL-10 [32], which is enhanced by the exposure to cerebrospinal fluid in the absence of any additional stimulation [34]. Primary astrocyte expression of both IL-10 mRNA and protein increases in response to LPS and TLR stimulation in a dose-dependent fashion [16,17,32]. It is also noteworthy that the fold-change in astrocyte production of IL-10 is greater than that in microglia when co-cultured, suggesting that astrocytes are more sensitive to stimuli that trigger IL-10 production [17]. In human postmortem brain tissue samples, IL-10, IL-4, and their receptors co-localize with reactive astrocytes in active demyelinating MS lesions, as well as in areas of brain infarction [35]. Perivascular astrocytes forming glial-scar-like barriers that restrict inflammatory adaptive immune cell infiltration exhibit prominent IL-10 expression in both human MS tissue and mouse EAE models [36,37,38].

Targeted overexpression of IL-10 by astrocytes also modifies the cellular responses to FNA. This increase in astrocytic IL-10 induces greater upregulation of IL-10R expression on FMN, suggesting a feed-forward role of IL-10 in the FMNuc that promotes its own signaling [30]. These data support findings from our laboratory showing that, while total IL-10 protein levels do not significantly change in the FMNuc after axotomy, IL-10R expression increases in a manner partially dependent on CD4+ T cell infiltration [1,9]. Another possible explanation for the apparent discrepancy between the unchanged IL-10 levels in the axotomized FMNuc and the induction of IL-10 production by astrocytes observed via IHC is that the amount of IL-10 produced by astrocytes may be small in comparison with that produced constitutively by neurons; alternatively, there may be a slight decrease in IL-10 production by neurons after axotomy (perhaps secondary to neuronal death) that is compensated for by astrocytes. Altogether, these data indicate roles for astrocyte production of IL-10 in modulating the immune response to injury or disease, as well as promoting neuroprotective IL-10 signaling in neurons. This immune modulation may take the form of an immunosuppressive role, as is observed in MS [35,36], or it may favor the infiltration of neuroprotective T cell subsets [30]. As astrocytes are both highly mobile and optimally situated for blood–brain barrier surveillance, a shift in IL-10 production toward the astrocyte suggests a more immunomodulatory role after axotomy, in comparison with constitutive IL-10 expression in neurons.

Microglial production of IL-10 after FNA remains ambiguous, as the results of this study suggest that microglia constitutively produce Il10 mRNA without evidence of protein translation. Production of IL-10 is heavily regulated on a post-transcriptional level, and many cell types express Il10 mRNA constitutively but do not translate IL-10 protein [39,40]. This may represent a mechanism by which microglia can rapidly synthesize IL-10 only after the correct stimulus. LPS [16,17], IFNγ [41], TLR stimulation [42], purinergic receptor activation [43], and T cell interactions via CD40 and CD80/86 [18] are all known to stimulate IL-10 production by microglia in vitro. LPS injection also stimulates microglial production of IL-10 in vivo, and blockade of this IL-10 signaling causes a significant increase in cortical neuron cell death [44]. IL-10 production by spinal cord parenchymal microglia is observed in rodent models of EAE [37], as well as in active MS lesions in humans [35], although the effect is not as robust as in astrocytes. These data suggest that, while microglia are capable of producing IL-10 in response to overt inflammatory stimuli in vitro and in vivo, the relatively mild inflammation induced by FNA may not be severe enough to trigger IL-10 production to a degree detectable by IHC.

To determine whether IL-10 production by neurons, astrocytes, or microglia is necessary for CD4+ T cell-mediated neuroprotection, a Cre/Lox system was employed to selectively knock out IL-10 production in each of these populations prior to axotomy. Selective deletion of IL-10 revealed that production by any of these single central sources was not critical for neuroprotection. It is unknown whether this selective IL-10 knockout affects CD4+ T cell recruitment or function in the FMNuc. This is an important limitation of this study, as CD4+ T cell responsiveness to IL-10 is necessary for their ability to confer neuroprotection [9]. After knockout, it is likely that the IL-10 remaining from one source is sufficient to mediate neuroprotection on its own; similarly, selectively knocking out IL-10 production from a single source may induce enhanced compensatory production from another. Both neuronal and astrocyte production of IL-10 in tandem may be necessary for FMN survival after axotomy, proposing a future direction for evaluating the impact of IL-10 knockout in both populations (as well as in triple knockouts of neurons, astrocytes, and microglia) simultaneously.

**Figure 8:**
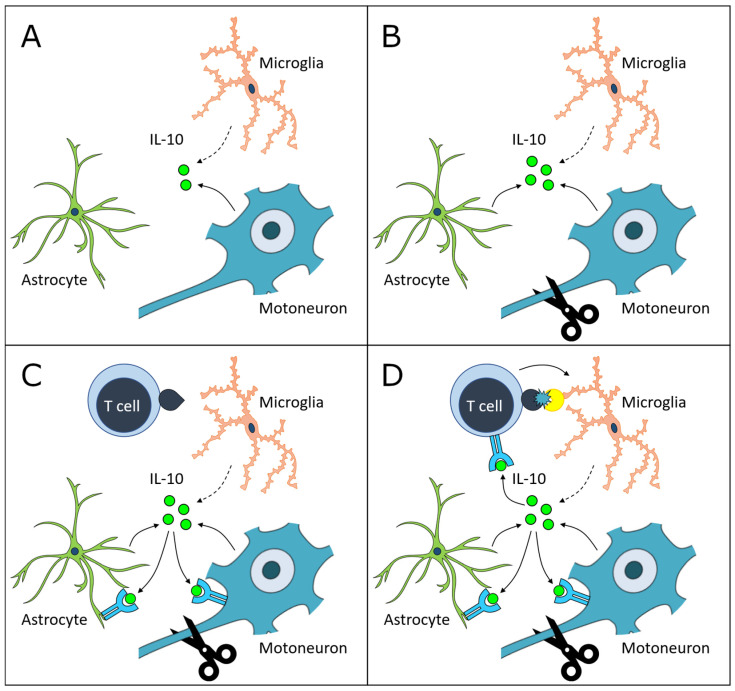
(**A**) Prior to FNA, FMN constitutively produces IL-10. Microglia constitutively transcribe *Il10* mRNA, but protein translation is unknown. (**B**) Astrocytic IL-10 production is axotomy-induced. (**C**) The appearance of CD4+ T cells in the FMNuc promotes upregulation of IL-10R expression on resident cells [1,9], which may provide trophic support to injured FMN. (**D**) CD4+ T cells must express the IL-10R to modulate microglial activation and confer neuroprotection [9].

In summary, these data support the dual roles for IL-10-mediated neuroprotection that involve (1) trophic support for injured FMN and (2) suppression of a neurotoxic immune response, which is proposed by our recent finding that CD4+ T cells that lack a functional IL-10R are incapable of mediating neuroprotection, perhaps through a failure to regulate appropriate microglial activation [9]. These roles are summarized in Figure 8 below. Constitutive neuronal expression of IL-10 may serve a homeostatic role for neuron resilience to stress by supporting neuron survival and synaptogenesis in an autocrine fashion [33,45,46]. Injury-induced IL-10 production by astrocytes may also provide trophic support to injured FMN and restrict harmful immune infiltration, as is observed in MS [35,36,38]. For IL-10 to perform these roles, its receptor must also be expressed on the necessary target cells. A direct trophic role of IL-10 is supported by the literature demonstrating IL-10R expression on neurons [1,30,31], and an indirect trophic role could be mediated via IL-10R expression on other FMNuc resident cells, potentially including astrocytes [1]. CD4+ T cells are required for the normal induction of IL-10R expression in the FMNuc after axotomy; furthermore, CD4+ T cells themselves must express functioning IL-10R to confer neuroprotection, and in the absence of this expression, promote a harmful autoinflammatory response to FNA [9]. These collective discoveries bridge two previously identified, but heretofore distinct, mechanisms of CD4+ T cell-mediated and IL-10-mediated FMN preservation into a single unified theory of neuroprotection after axotomy.

## Figures and Tables

**Table 1:** Mouse strains utilized in this study.

Strain (Jackson Strain No.)	Alternately Named	Source
B6(Cg)-Il10^tm1.1Karp^/J (014530)	IL-10/GFP reporter	The Jackson Laboratory
B6.129P2(Cg)-Cx3cr1^tm2.1(cre/ERT2)^Litt/WganJ (021160)	*Cx3cr1* ^Cre^	The Jackson Laboratory
B6.129P2-Il10^tm1Cgn^/J (002251)	IL-10^−/−^	The Jackson Laboratory
B6.Cg-Tg(GFAP-cre/ERT2)505Fmv/J (012849)	*Gfap* ^Cre^	The Jackson Laboratory
C57BL/6J (000664)	WT	The Jackson Laboratory
IL10^flox/flox^		Dr. Gang Huang, Cincinnati Children’s Hospital; see [10]
Tg(Thy1-cre/ERT2,-EYFP)HGfng/PyngJ (012708)	*Thy1* ^Cre^	The Jackson Laboratory

**Table 2:** Primer sequences for PCR.

Gene	5′ Primer Sequence	3′ Primer Sequence
IL10 exon 1 (133 bp in WT)	ATG CCT GGC TCA G	CCA CAT GCT CCT AGA GCT GC
IL10 floxed sequence and exon 2 (480 bp WT, 514 bp floxed)	CCA GCA TAG AGA GCT TGC ATT ACA	GAG TCG GTT AGC AGT ATG TTG TCC AG
*Thy1*^Cre^ transgene (300 bp)	TCT GAG TGG CAA AGG ACC TTA GG	CGC TGA ACT TGT GGC CGT TTA CG
*Thy1*^Cre^ internal control (200 bp)	CAA ATG TTG CTT GTC TGG TG	GTC AGT CGA GTG CAC AGT TT
*Gfap*^Cre^ transgene (200 bp)	GCC AGT CTA GCC CAC TCC TT	TCC CTG AAC ATG TCC ATC AG
*Gfap*^Cre^ internal control (324 bp)	CTA GGC CAC AGA ATT GAA AGA TCT	GTA GGT GGA AAT TCT AGC ATC ATC C
*Cx3cr1*^Cre^ transgene (300 bp)	AAG ACT CAC GTG GAC CTG CT	CGG TTA TTC AAC TTG CAC CA
*Cx3cr1*^Cre^ internal control (695 bp)	AAG ACT CAC GTG GAC CTG CT (common)	AGG ATG TTG ACT TCC GAG TTG

**Table 3:** Antibodies utilized for immunohistochemistry.

Antibody	Manufacturer and Cat. No.	Dilution
Mouse anti-GFAP 594 (used in IL-10/GFP reporter)	Thermo Fisher A-21295	1:500
Rabbit anti-GFP 488	Thermo Fisher A-21311	1:100
Rabbit anti-IBA1	Thermo Fisher 019-19741	1:500
Mouse anti-NeuN 555	Millipore MAB377A5	1:200
Rabbit anti-GFAP (used in perfused WT tissue)	Thermo Fisher PA3-16727	1:1000
Goat anti-IL-10	R&D Systems AF519	1:200
Donkey anti-rabbit 568 (used for GFAP, IBA1)	Abcam ab175470	1:1000
Donkey anti-goat 488 (used for IL-10)	Abcam ab150129	1:1000

## Data Availability

The datasets used during the study are available from the corresponding author upon reasonable request.

## Supplementary Materials

The following are available online at https://www.mdpi.com/article/10.3390/cells11193167/s1, Figure S1: Timelines for experiments in relation to axotomy at day 0. cKO = conditional knockout, IHC = immunohistochemistry, FMN = facial motoneuron.
