# Supplementary material for: Cellular Sources and Neuroprotective Roles of Interleukin-10 in the Facial Motor Nucleus after Axotomy
Source: Cells. 2022 Oct 9;11(19):3167. doi: 10.3390/cells11193167 (PMC9564302; doi:10.3390/cells11193167)
Supplement: Supplementary file 1 [file cells-11-03167-s001.zip › cells-1738121-Figure S1.pdf]

cells

MDPI

# Supplementary Materials

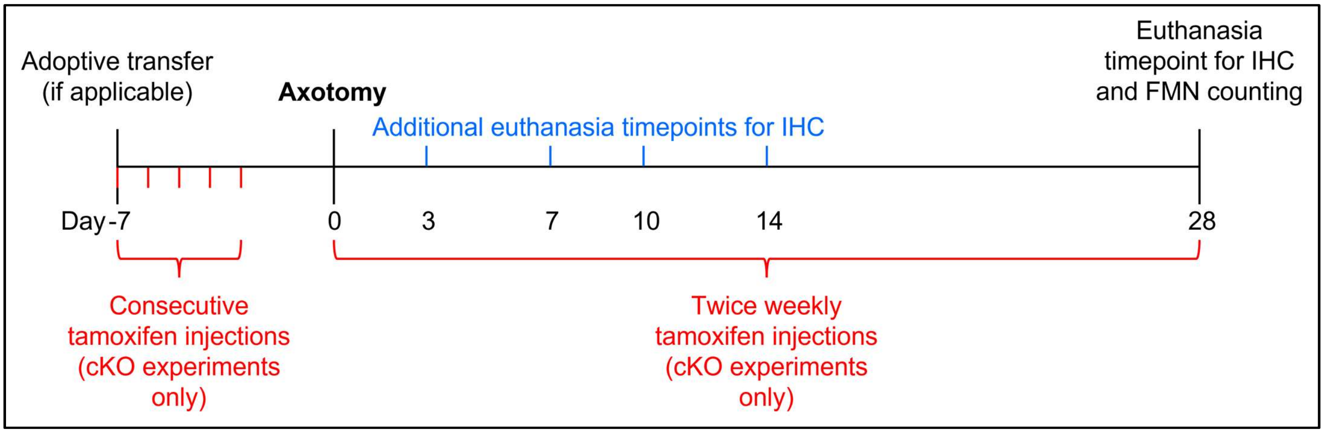


**Figure S1**. Timelines for experiments in relation to axotomy at day 0. *cKO* = conditional knockout, *IHC* = immunohistochemistry, *FMN* = facial motoneuron
